# Intracellular cAMP Signaling Pathway via G_s_ Protein-Coupled Receptor Activation in Rat Primary Cultured Trigeminal Ganglion Cells

**DOI:** 10.3390/biomedicines11092347

**Published:** 2023-08-23

**Authors:** Yuki Kunioku, Maki Kimura, Takehito Ouchi, Kenichi Fukuda, Yoshiyuki Shibukawa

**Affiliations:** 1 Department of Physiology, Tokyo Dental College, 2-9-18, Kanda-Misaki-cho, Chiyoda-ku, Tokyo 101-0061, Japan; kuniokuyuki@tdc.ac.jp (Y.K.); takehitoo@tdc.ac.jp (T.O.); yshibuka@tdc.ac.jp (Y.S.); 2 Division of Special Needs Dentistry and Orofacial Pain, Department of Oral Health and Clinical Science, Tokyo Dental College, 2-9-18, Kanda-Misaki-cho, Chiyoda-ku, Tokyo 101-0061, Japan; kfukuda@tdc.ac.jp

**Keywords:** trigeminal ganglion neurons, rat, Gα_s_ protein-coupled receptors, adenosine 3′,5′-cyclic monophosphate, adenylyl cyclase, primary culture

## Abstract

G protein-coupled receptors in trigeminal ganglion (TG) neurons are often associated with sensory mechanisms, including nociception. We have previously reported the expression of P2Y_12_ receptors, which are G_i_ protein-coupled receptors, in TG cells. Activating P2Y_12_ receptors decreased the intracellular free Ca^2+^ concentration ([Ca^2+^]_i_). This indicated that intracellular adenosine 3′,5′-cyclic monophosphate (cAMP) levels can mediate Ca^2+^ signaling in TG cells. Here, we report more extensive-expression patterns of G_s_ protein-coupled receptors in primary cultured TG neurons isolated from 7-day-old newborn Wistar rats and further examine the roles of these receptors in cAMP signaling using the BacMam sensor in these neurons. To identify TG neurons, we also measured [Ca^2+^]_i_ using fura-2 in TG cells and measured intracellular cAMP levels. TG neurons were positive for Gα_s_ protein-coupled receptors, beta-2 adrenergic (β_2_), calcitonin gene-related peptide (CGRP), adenosine A_2A_ (A_2A_), dopamine 1 (D1), prostaglandin I_2_ (IP), and 5-hydroxytriptamine 4 (5-HT_4_) receptor. Application of forskolin (FSK), an activator of adenylyl cyclase, transiently increased intracellular cAMP levels in TG neurons. The application of a phosphodiesterase inhibitor augmented the FSK-elicited intracellular cAMP level increase. These increases were significantly suppressed by the application of SQ22536, an adenylyl cyclase inhibitor, in TG neurons. Application of agonists for β_2_, CGRP, A_2A_, D1-like, IP, and 5-HT_4_ receptors increased intracellular cAMP levels. These increases were SQ22536-sensitive. These results suggested that TG neurons express β_2_, CGRP, A_2A_, D1, IP, and 5-HT_4_ receptors, and the activations of these Gα_s_ protein-coupled receptors increase intracellular cAMP levels by activating adenylyl cyclase.

## 1. Introduction

Quantitative and qualitative changes in sensory-related and pain-related receptors in peripheral nerves cause orofacial sensations, including pain. Plasma membrane receptors associated with sensory perception are classified into G protein-coupled receptors (GPCRs) (metabotropic receptors) and ionotropic receptors (ligand-gated ion channels). GPCRs are seven-transmembrane receptors coupled with heterotrimeric G proteins [1]. The G proteins are constituted by α, β, and γ subunits [1]. The Gα subunits are typically classified into four classes based on their functional and genetic differences: Gα_s_, Gα_i_, Gα_q_, and Gα_12/13_ [2]. Gα_s_ and Gα_i_ are involved in adenylyl cyclase activity, which regulates intracellular adenosine 3′,5′-cyclic monophosphate (cAMP) synthesis from adenosine triphosphate (ATP) degradation [3]. The binding of the ligands to Gα_s_ protein-coupled receptors activates adenylyl cyclase, resulting in the generation of cAMP [4]. cAMP is an intracellular signal transmitter that acts as a second messenger and activator of cAMP-dependent protein kinase A (PKA). PKA participates in a variety of physiological processes, such as vascular smooth muscle contraction, gastrointestinal motility, bronchial smooth muscle contraction, and endocrine and exocrine enhancement [5]. The cAMP generated by adenylyl cyclase is hydrolyzed into adenosine monophosphate (5′-AMP) by phosphodiesterase (PDE) enzymes. When ligands bind to Gα_i_ protein-coupled receptors, adenylyl cyclase activity is suppressed, resulting in decreased intracellular cAMP production [4].

In the trigeminal ganglion (TG), the cell bodies of the primary nociceptive neurons that innervate the orofacial region are localized. Gα_s/i_ protein-coupled receptors are currently attracting attention as target plasma membrane proteins for many analgesics [6]. Pan et al. reported that almost all GPCR agonists, which are coupled to Gα_i_ proteins, have analgesic action [7]. Previous studies have demonstrated the expression of P2Y_12_ receptors, which are Gα_i_ protein-coupled receptors, in rat TG cells and suggested that a decrease in intracellular cAMP level by receptor activation may regulate the intracellular Ca^2+^ mobilization pathway [8]. However, there are few studies on the functional expression of Gα_s_ protein-coupled receptors, and the detailed intracellular cAMP signaling pathway that is induced by the activation of the receptors in TG neurons remains unknown. Therefore, this study investigated the functional expression of Gα_s_ protein-coupled beta-2 adrenergic (β_2_) receptor, adenosine A_2A_ (A_2A_) receptor, calcitonin gene-related peptide (CGRP) receptor, dopamine 1 (D1) receptor, prostaglandin I_2_ (IP) receptor, and 5-hydroxytriptamine 4 (5-HT_4_) receptor in primary cultured rat TG neurons.

## 2. Materials and Methods

### 2.1. Isolation and Primary Culture of TG Cells

We followed the Guiding Principles for the Care and Use of Animals in the Field of Physiological Sciences approved by the American Physiological Society and the Council of the Physiological Society of Japan to care for all animals. We also followed the guidelines established by the National Institutes of Health (Bethesda, MD, USA) concerning the use and care of animals for experimental procedures. The Ethics Committee of Tokyo Dental College approved this study (approval Nos. 200301 and 210301).

We anesthetized newborn Wistar rats (7 days old) with pentobarbital sodium (50 mg/kg intraperitoneally) following isoflurane inhalation (3.0% vol) and obtained TG cells. The TG cells were isolated enzymatically by 20 U/mL papain (Worthington Biochemical Co., Lakewood, NJ, USA) with Hank’s balanced salt solution (HBSS) (0.34 mM Na_2_HPO_4_, 0.44 mM KH_2_PO_4_, 0.5 mM MgCl_2_, 2.0 mM CaCl_2_, 4.17 mM NaHCO_3_, 5.0 mM KCl, 5.55 mM glucose, and 137 mM NaCl [pH 7.4 Tris]) for 20 min at 37 °C, then dissociated by trituration [9,10]. We carried out primary culture of TG cells in Leibovitz’s L-15 medium (Life Technologies Co., Grand Island, NY, USA), including 10% fetal bovine serum (Life Technologies Co.), 1% penicillin-streptomycin (Life Technologies Co.), 1% amphotericin B (Sigma-Aldrich Co., St. Louis, MO, USA), 30 mM glucose, and 24 mM NaHCO_3_ (pH 7.4). We measured intracellular cAMP levels and intracellular free Ca^2+^ concentration ([Ca^2+^]_i_) in primary cultured TG cells, which were maintained in culture for 48–60 h after isolation. Briefly, after 24 h of isolation, we replaced the medium with a fresh one containing “cADDis” (see below), and then incubated the cells for 24–36 h before using them for the experiments. TG cells used for immunostaining were cultured for 36 h after isolation without any medium change. These cells were cultured and maintained at 37 °C in an incubator filled with 95% air and 5% CO_2_ [8].

### 2.2. Immunofluorescence Microscopy

We cultured isolated rat TG cells in eight-well glass chambers (Iwaki, Shizuoka, Japan, 5732-008) at 37 °C in a 5% CO_2_ incubator for 36 h without changing the medium. We fixed the TG cells with paraformaldehyde (4%) (FUJIFILM Wako Pure Chemical Co., Osaka, Japan) and rinsed them with phosphate-buffered saline (PBS; Life Technologies Co.). We incubated with blocking buffer (Blocking One; Nacalai Tesque, Kyoto, Japan) for 10–15 min at room temperature, then applied primary antibodies overnight at 4 °C or for 3–4 h at room temperature: rabbit monoclonal anti-β-2 adrenergic receptor (1:200; MAB10040; 2204C; R&D systems, Minneapolis, MN, USA), mouse monoclonal anti-adenosine A_2A_-receptor (1:200; sc-32261; 7F6-G5-A2; Santa Cruz Biotechnology, Inc., Dallas, TX, USA), mouse monoclonal anti-D1 dopamine receptors (1:200; sc-33660; SG2-D1a; Santa Cruz Biotechnology, Inc.), rabbit monoclonal anti-calcitonin receptor-like receptor (CALCRL) (1:200; 703811; 8H9L8; Life Technologies Co.), mouse monoclonal anti-IP receptor (1:200; sc-365268; B-3; Santa Cruz Biotechnology, Inc.), rabbit polyclonal anti-heterotrimeric G-protein α-subunit Gα_s_ (Gnas) (1:200; A5546; ABclonal, Tokyo, Japan), rabbit polyclonal anti-5HT_4_ receptor (1:200; bs-2127R; Bioss, Woburn, MA, USA), and mouse monoclonal anti-neurofilament heavy chain (NF-H) (1:200; sc-32729; RNF402; Santa Cruz Biotechnology, Inc.). The cells were then incubated with secondary antibodies of Alexa Fluor^®^ 488 donkey anti-mouse (#A21202), Alexa Fluor 568 donkey anti-mouse (#A10037), and Alexa Fluor^®^ 488 donkey anti-rabbit (#A21206) (all from Invitrogen) for 60 min at room temperature. A mounting medium with 4′,6-diamidino-2-phenylindole (DAPI; Abcam, Cambridge, UK) was dropped on the stained samples. DAPI was used for staining nuclei. We observed images of immunofluorescence staining using a fluorescence microscope (BZ-X710; Keyence, Osaka, Japan).

### 2.3. Intracellular cAMP Level Assays in Living Cells

For monitoring cAMP dynamics in living cells, we changed to the medium containing 0.4% Na-Butyrate and 16.7% BacMam sensor (green upward cAMP difference detector in situ [cADDis]; Montana Molecular, Bozeman, MT, USA) after 24 h of isolation. The TG cells were incubated in the medium at 37 °C for 24–36 h and then washed with HBSS. BacMam sensor-transfected TG cells were observed using a microscope (IX73, Evident Co., Tokyo, Japan), which equipped an intensified charge-coupled device camera system, an excitation wavelength selector, and an HCImage system (Ver. 4.3.1, Hamamatsu Photonics, Shizuoka, Japan). cADDis fluorescence emission (F_506_) was measured at 517 nm in response to an excitation wavelength of 506 nm. The intracellular cAMP level was represented as the fluorescence ratio (F/F_0_) of the F_506_ value (F) to the resting value (F_0_). The intracellular cAMP level was measured from TG cells in the designated measurement field (Region of Interest: ROI) that was randomly chosen in the culture dish with cADDis-transfected cells. All experiments were performed at room temperature (28 °C).

### 2.4. Measurement of Intracellular Free Ca^2+^ Concentration

We loaded fura-2 acetoxymethyl ester (10 μM) (Dojindo, Kumamoto, Japan) (with pluronic acid F-127 (0.1% (*w*/*v*)); Life Technologies Co.) into cADDis-transfected primary cultured TG cells in HBSS for 90 min at 37 °C. We then rinsed the cultured TG cells with fresh HBSS and put them on the culture dish placed on a microscope stage (IX73, Evident Co.). We measured fura-2 fluorescence emission at 510 nm by alternating excitation wavelengths of 340 nm (F340) and 380 nm (F380) (HCImage software (Ver. 4.3.1); Hamamatsu Photonics). The software controls an intensified charge-coupled device camera system (Hamamatsu Photonics) and a selector for excitation wavelength. In TG cells tested for intracellular cAMP level measurement, we measured [Ca^2+^]_i_ using the fluorescence ratio of F340 to F380 (R_F340/F380_) at two excitation wavelengths. The changes in [Ca^2+^]_i_ were described in F/F_0_ units; the R_F340/F380_ value (F) was normalized to the resting value (F_0_). We performed all the experiments at room temperature (28 °C).

### 2.5. Solutions and Reagents

HBSS was used as a standard extracellular solution. In brightfield images of primary cultured rat TG cells, we were unable to discriminate the neurons from the satellite glial cells (SGCs). A high K^+^ solution (0.34 mM Na_2_HPO_4_, 0.44 mM KH_2_PO_4_, 0.5 mM MgCl_2_, 2.0 mM CaCl_2_, 4.17 mM NaHCO_3_, 5.55 mM glucose, 50 mM KCl, and 91 mM NaCl; pH 7.4) activates membrane depolarization-elicited elevations in the [Ca^2+^]_i_ in TG neurons. TG neurons were distinguished from glial cells in TG cells by applying a high K^+^ solution [9]. The D1-like receptor agonist SKF83959 was purchased from Cayman Chemical (Ann Arbor, MI, USA). The β_2_ receptor agonist isoproterenol (ISO) was obtained from Montana Molecular. All other reagents were obtained from Tocris Bioscience (Bristol, UK), except where indicated. Stock solutions were prepared by dissolving the reagents in dimethyl sulfoxide (DMSO) for forskolin (FSK), IBMX, SQ22536, PSB0777, SKF83959, beraprost, and BIMU8 and in MilliQ water for CGRP (rat). Stock solutions were diluted with standard solutions to the appropriate concentrations before use. For both intracellular cAMP and Ca^2+^ measurements, we applied a standard extracellular solution with or without each receptor agonist, enzyme activator, or inhibitor, as well as a high K^+^ solution, through superfusion using a rapid gravity-fed perfusion system (AutoMate Scientific, Berkeley, CA, USA). We applied a series of repeated applications (every 1 min) of each receptor agonist, enzyme activator, or inhibitor to the cells and rinsed with standard extracellular solution until the F/F_0_ value returned to baseline. Solution changes were completed within 20 ms.

### 2.6. Statistical and Offline Analysis

In [Ca^2+^]_i_ and intracellular cAMP level measurements, data are represented as the mean ± standard error (S.E.) of *n* observations. The *n* shows the number of independent experiments. In the analysis of cell sizes, data are represented as the mean ± standard deviation (S.D.) of *n* observations. The *n* shows the number of cells. We analyzed the data using the nonparametric Friedman test and Dunn’s multiple comparison test. Parametric statistical significance was determined by one-way ANOVA with Tukey’s post-hoc test. We set statistical significance at *p* < 0.05. Statistical analysis was carried out using GraphPad Prism 7.0 (GraphPad Software, La Jolla, CA, USA).

## 3. Results

### 3.1. Immunolocalization of Gnas and NF-H in the TG Neurons

**Figure 1:**
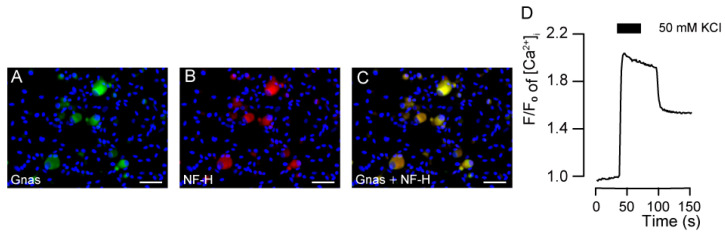
Immunolocalization of Gnas and neurofilament heavy chain (NF-H), as well as a representative trace of increased intracellular free Ca^2+^ concentration ([Ca^2+^]_i_) evoked by membrane depolarization in primary cultured trigeminal ganglion (TG) neurons. (**A**,**B**) Primary cultured TG neurons showed positive immunoreactivity to the Gnas (green in (**A**)) and NF-H, a marker of A-neuron (red in (**B**)). (**C**) These Gnas immunoreactivities colocalized with neurons positive for NF-H. Blue shows Nuclei. Scale bar; 50 μm. (**D**) Representative trace of transient [Ca^2+^]_i_ increased response to high K^+^ solution in the presence of extracellular Ca^2+^. The black box indicates the time period in which a high K^+^ solution was applied.

The cultured TG neurons were immunopositive for rabbit monoclonal anti-heterotrimeric Gnas (Figure 1A) and mouse monoclonal anti-NF-H (Figure 1B). We observed Gnas immunoreactivity in the cell bodies of primary cultured TG cells. The Gnas immunoreactivity mainly colocalized with neurons positive for NF-H, a marker of neurons with myelinated primary-afferent A fibers (A-neuron) (Figure 1C).

### 3.2. Forskolin-Induced Intracellular cAMP Increases Were Sensitive to Inhibitors of Adenylyl Cyclase and Phosphodiesterase

The TG cells (*n* = 70; total numbers of independent experiments) tested for intracellular cAMP level measurement showed [Ca^2+^]_i_ increases by applying extracellular high K^+^-induced membrane depolarization. The peak value was 2.18 ± 0.07 F/F_0_ units. This result indicated that the cells showing an increase in intracellular [Ca^2+^]_i_ in response to membrane depolarization were neuronal populations (Figure 1D). We then analyzed intracellular cAMP signaling specifically in these neuronal cell populations.

**Figure 2:**
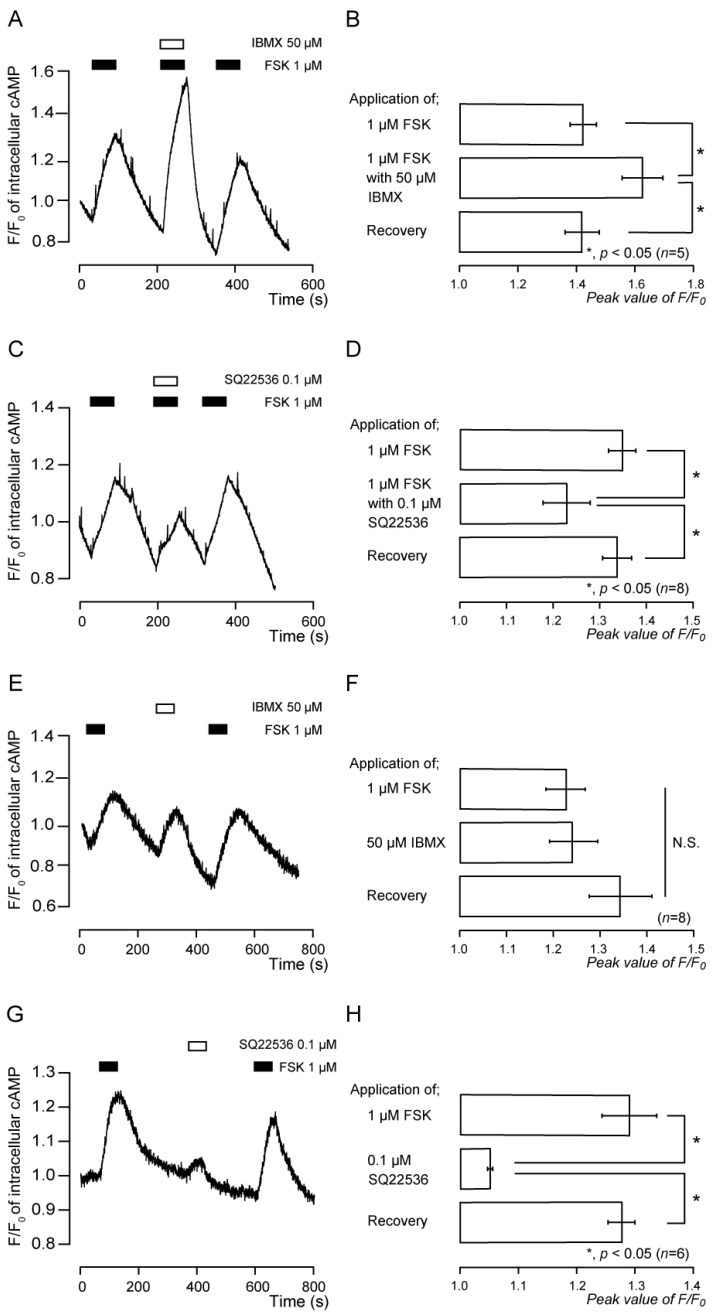
Increases in adenylyl cyclase activator-induced intracellular cAMP level, and effect of phosphodiesterase and adenylyl cyclase inhibitors on these increases in TG neurons. (**A**,**C**) Representative traces of FSK-induced transient increases in intracellular cAMP level in the presence of extracellular Ca^2+^. Black boxes denote time periods of 1 μM FSK addition to the external solution. White boxes indicate the time of addition of IBMX (**A**) or SQ22536 (**C**) to the external solution. (**B**,**D**) Summary bar graphs of FSK-evoked increases in intracellular cAMP level, with (middle column) or without (upper column) 50 µM IBMX (**B**) or 0.1 µM SQ22536 (**D**), in the presence of extracellular Ca^2+^. Each recovery (lower column) indicates that the effects of IBMX (**B**) and SQ22536 (**D**) are reversible. (**E**,**G**) Representative traces of FSK-, IBMX-, or SQ22536-induced transient increases in intracellular cAMP level in the presence of extracellular Ca^2+^. Black boxes denote time periods of 1 μM FSK addition to the external solution. White boxes indicate the time of addition of IBMX (**E**) or SQ22536 (**G**) to the external solution. (**F**,**H**) Summary bar graphs of FSK- (upper columns in (**F**,**H**), IBMX- (middle column in (**F**)), or SQ22536- (middle column in (**H**)) induced intracellular cAMP level increase in the presence of extracellular Ca^2+^. Each bar shows mean ± standard error (S.E.) of five (**B**), eight (**D**), eight (**F**), and six (**H**) experiments. Asterisk: * *p* < 0.05 indicates statistically significant differences between columns (indicated by a solid line). N.S. represents that there is no significance between columns.

Application of FSK (1 μM), an adenylyl cyclase activator, transiently increased intracellular cAMP levels (Figure 2A–D). The peak values were 1.42 ± 0.04 F/F_0_ units (*n* = 5; Figure 2B) and 1.37 ± 0.03 F/F_0_ units (*n* = 8; Figure 2D). FSK-induced intracellular cAMP level increases were augmented by the application of a PDE inhibitor, IBMX (50 μM) (Figure 2A,B). The peak value was 1.63 ± 0.07 F/F_0_ units (*n* = 5; Figure 2B). SQ22536 (0.1 µM), an adenylyl cyclase inhibitor, significantly and reversibly inhibited the increase in FSK-induced intracellular cAMP levels in TG cells (Figure 2C,D). We also investigated the effects of the independent application of IBMX or SQ22536 on intracellular cAMP levels in TG cells. The application of IBMX increased the intracellular cAMP level (Figure 2E,F). The peak value was 1.24 ± 0.05 F/F_0_ units (*n* = 8) (Figure 2F); there are no significant differences in intracellular cAMP elevation between the IBMX-induced and FSK-induced ones. These results suggest that intracellular cAMP level elevation induced by simultaneous application of IBMX and FSK (middle column in Figure 2B) contained two components: intracellular cAMP level increase evoked by (1) adenylyl cyclase activation (upper column in Figure 2B,F) and (2) PDE inhibition (middle column in Figure 2F). SQ22536 slightly increased intracellular cAMP levels in TG cells (Figure 2G,H). The peak value was 1.05 ± 0.004 F/F_0_ units (*n* = 6) (Figure 2H). Nevertheless, the application of SQ22536 significantly inhibited FSK-induced intracellular cAMP level increases in Figure 2C,D, suggesting that the effect of SQ22536 itself on intracellular cAMP level was negligible.

### 3.3. TG neurons Are Immunopositive for Gnas as well as Gα_s_ Protein-Coupled Receptor Antibodies

**Figure 3:**
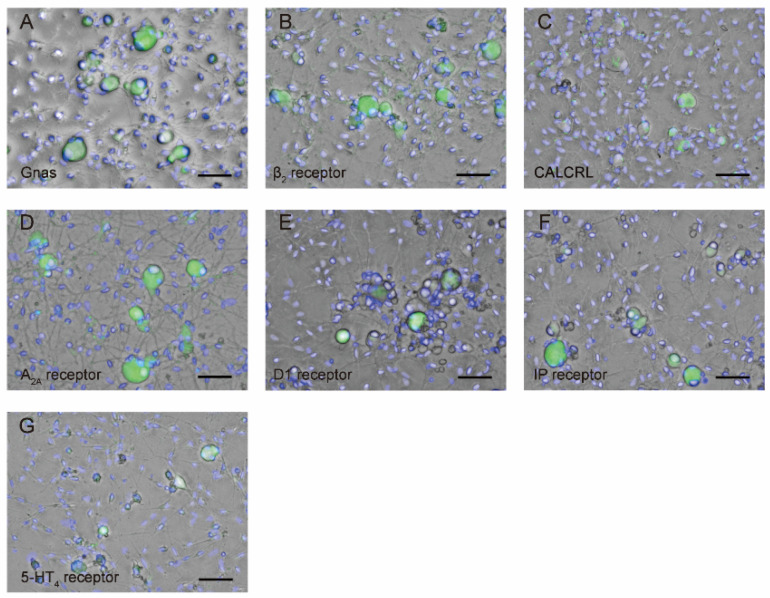
Immunofluorescence analyses of Gnas (**A**), beta-2 adrenergic (β_2_) receptor (**B**), calcitonin receptor-like receptor (CALCRL) (**C**), adenosine A_2A_ (A_2A_) receptor (**D**), dopamine 1 (D1) receptor (**E**), prostaglandin I_2_ (IP) receptor (**F**), and 5-hydrotriptamine 4 (5-HT_4_) receptor (**G**) in primary cultured TG cells are shown (green). Blue shows nuclei. Scale bar: 50 μm.

TG cells were immunopositive for the Gnas protein (Figure 3A) as well as the β_2_ receptor (Figure 3B), CALCRL (Figure 3C), A_2A_ (Figure 3D), D1 (Figure 3E), IP (Figure 3F), and 5-HT_4_ (Figure 3G) receptors. Note that the CGRP receptor is composed of CALCRL and receptor activity modifying protein 1 (RAMP1). We measured the longest cell diameters as the parameter showing the size of TG cells. The average diameters of TG cells showing immunopositives were 27.1 ± 7.3 μm for Gnas (*n* = 19), 27.1 ± 5.0 μm for the β_2_ receptor (*n* = 11), 23.2 ± 8.7 μm for CALCRL (*n* = 14), 27.1 ± 9.3 μm for the A_2A_ receptor (*n* = 13), 30.1 ± 7.6 μm for the D1 receptor (*n* = 6), 26.9 ± 6.9 μm for the IP receptor (*n* = 10), and 27.5 ± 8.2 μm for the 5-HT_4_ receptor (*n* = 18).

### 3.4. Functional Expression of β_2_ Receptors in TG Neurons

**Figure 4:**
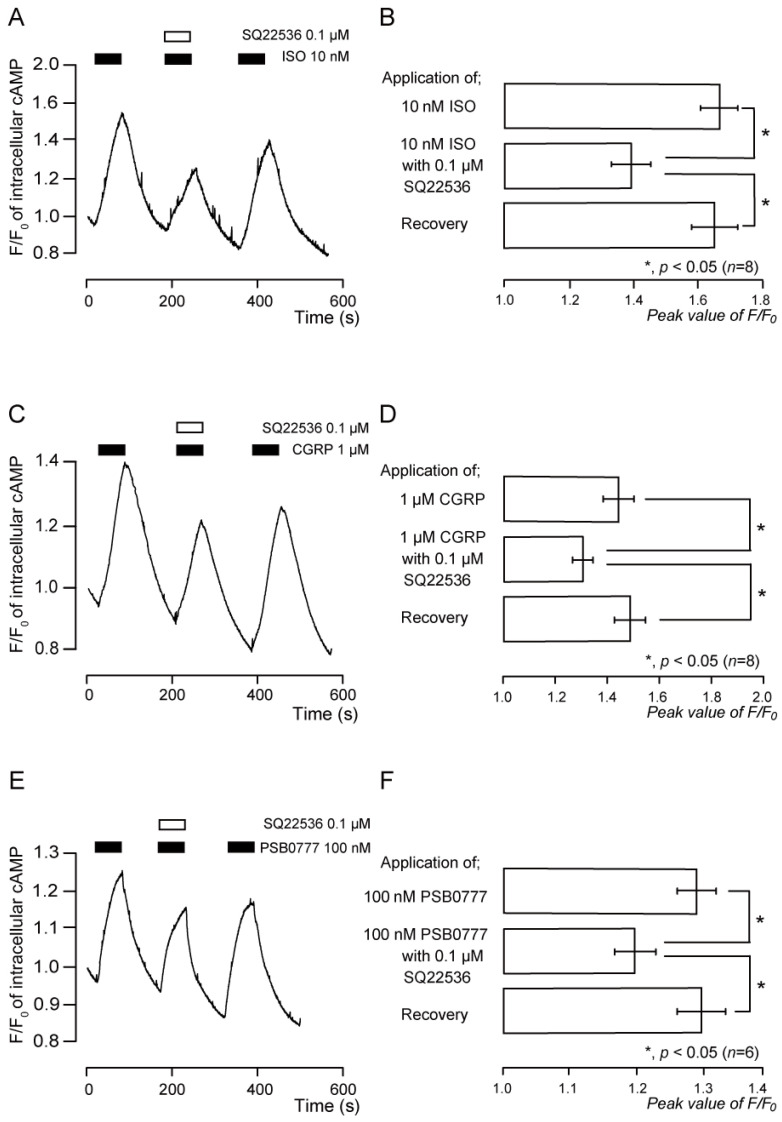
Intracellular cAMP level increases by the β_2_, CGRP, and A_2A_ receptor agonists, which were suppressed by the adenylyl cyclase inhibitor. (**A**,**C**,**E**) Representative traces of transient increases in intracellular cAMP level in response to 10 nM isoproterenol (ISO) (**A**), 1 µM CGRP (rat) (**C**), and 100 nM PSB0777 (**E**) without or with 0.1 µM SQ22536 in the presence of extracellular Ca^2+^ (2.5 mM). Black boxes indicate time periods of ISO (**A**), CGRP (rat) (**C**), and PSB0777 (**E**) addition to the extracellular solution. White boxes denote the time of addition of SQ22536 to the external solution. (**B**,**D**,**F**) Summary bar graphs of ISO- (**B**), CGRP (rat)- (**D**), and PSB0777- (**F**) induced intracellular cAMP level increases, with (middle columns) or without (upper columns) 0.1 µM SQ22536 in the presence of extracellular Ca^2+^. Each recovery (lower columns) indicates that SQ22536 acts reversibly. Each bar shows mean ± S.E. of eight (**B**), eight (**D**), and six (**F**) experiments. Asterisk: * *p* < 0.05 shows statistically significant differences between columns (indicated by a solid line).

Application of ISO (10 nM), an agonist of the β_2_ receptors, induced transient increases in intracellular cAMP levels (Figure 4A,B). The peak value was 1.66 ± 0.05 F/F_0_ units (*n* = 8; Figure 4B). SQ22536 (0.1 µM) significantly and reversibly inhibited the increase in intracellular cAMP levels induced by ISO (Figure 4A,B). The peak value was 1.39 ± 0.06 F/F_0_ units (*n* = 8; Figure 4B).

### 3.5. Functional Expression of CGRP Receptors in TG Neurons

Application of CGRP (rat) (1 µM), an agonist of the CGRP receptors, transiently increased intracellular cAMP levels (Figure 4C,D). The peak value was 1.43 ± 0.06 F/F_0_ units (*n* = 8; Figure 4D). SQ22536 (0.1 µM) significantly and reversibly inhibited the increase in intracellular cAMP level evoked by CGRP (rat) (Figure 4C,D). The peak value was 1.30 ± 0.04 F/F_0_ units (*n* = 8; Figure 4D).

### 3.6. Functional Expression of A_2A_ Receptors in TG Neurons

Application of PSB0777 (100 nM), an agonist of the A_2A_ receptors, transiently increased intracellular cAMP levels (Figure 4E,F). The peak value was 1.30 ± 0.03 F/F_0_ units (*n* = 6; Figure 4F). SQ22536 (0.1 µM) significantly and reversibly inhibited PSB0777-induced intracellular cAMP level increases (Figure 4E,F). The peak value was 1.20 ± 0.03 F/F_0_ units (*n* = 6; Figure 4F).

### 3.7. Functional Expression of D1-like Receptors in TG Neurons

**Figure 5:**
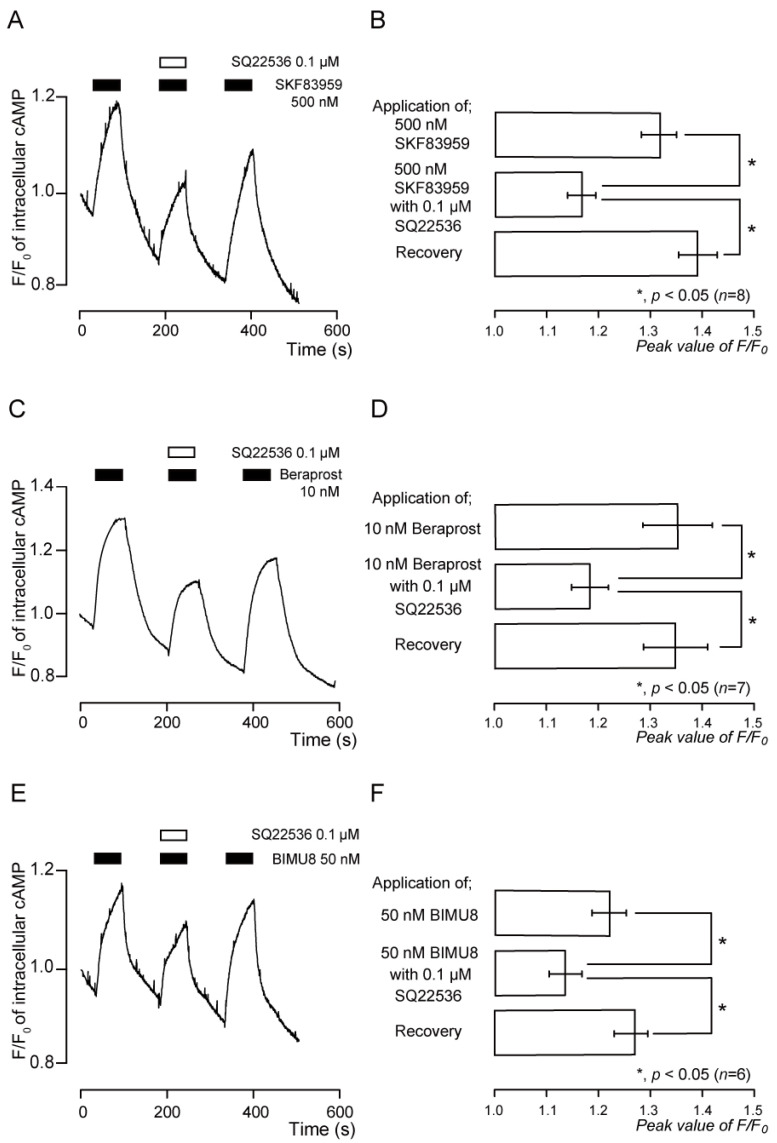
Intracellular cAMP level increases by the D1-like, IP, and 5-HT_4_ receptor agonists, which were suppressed by adenylyl cyclase inhibition. (**A**,**C**,**E**) Representative traces of transient intracellular cAMP level increases in response to 500 nM SKF83959 (**A**), 10 nM beraprost (**C**), and 50 nM BIMU8 (**E**) with or without 0.1 µM SQ22536 in the presence of extracellular Ca^2+^ (2.5 mM). Black boxes indicate time periods of application for SKF83959 (**A**), beraprost (**C**), and BIMU8 (**E**) to the external solution. White boxes denote the time of addition of SQ22536 to the external solution. (**B**,**D**,**F**) Summary bar graphs of SKF83959- (**B**), beraprost- (**D**), and BIMU8- (**F**) induced intracellular cAMP level increases, without (upper columns) or with (middle columns) 0.1 µM SQ22536 in the presence of extracellular Ca^2+^. Each recovery (lower columns) indicates that SQ22536 acts reversibly. Each bar denotes mean ± S.E. of eight (**B**), seven (**D**), and six (**F**) experiments. Asterisk: * *p* < 0.05 shows statistically significant differences between columns (indicated by a solid line).

Application of SKF83959 (500 nM), an agonist of the D1-like receptors, transiently increased intracellular cAMP levels (Figure 5A,B). The peak value was 1.32 ± 0.03 F/F_0_ units (*n* = 8; Figure 5B). SQ22536 (0.1 µM) significantly and reversibly inhibited SKF83959-induced intracellular cAMP levels, which increased to a peak value of 1.17 ± 0.02 F/F_0_ units (*n* = 8; Figure 5B).

### 3.8. Functional Expression of IP Receptors in TG Neurons

The cell surface receptor for PGI_2_ is the IP receptor, which is a GPCR. Application of beraprost (10 nM), an agonist of the IP receptor, transiently increased intracellular cAMP levels (Figure 5C,D). The peak value was 1.35 ± 0.04 F/F_0_ units (*n* = 7; Figure 5D). SQ22536 (0.1 µM) significantly and reversibly inhibited beraprost-induced intracellular cAMP level increases (Figure 5C,D). The peak value was 1.18 ± 0.03 F/F_0_ units (*n* = 7; Figure 5D).

### 3.9. Functional Expression of 5-HT_4_ Receptors in TG Neurons

Application of BIMU8 (50 nM), an agonist of the 5-HT_4_ receptors, transiently increased intracellular cAMP levels (Figure 5E,F). The peak value was 1.22 ± 0.03 F/F_0_ units (*n* = 6; Figure 5F). SQ22536 (0.1 µM) significantly and reversibly inhibited BIMU8-induced intracellular cAMP level increases (Figure 5E,F). The peak value was 1.13 ± 0.03 F/F_0_ units (*n* = 6; Figure 5F).

## 4. Discussion

In this study, we showed that TG neurons functionally express Gα_s_ protein-coupled receptors, namely, β_2_, CGRP, A_2A_, D1, IP, and 5-HT_4_ receptors. Activation of these receptors stimulates the activation of adenylyl cyclase, resulting in an intracellular cAMP level increase in TG neurons. The average of the longest diameters of Gnas-, and β_2_, A_2A_, D1, IP, and 5HT_4_ receptor-immunopositive cells ranged from 26.9 µm to 30.1 µm. The average of the longest diameters of Gnas, β_2_, A_2A_, D1, IP, and 5HT_4_, except CALCRL, receptor-immunopositive cells, was 27.4 ± 7.4 µm. Although we used primary cultured TG neurons, based on previous TG neuron classification studies, acutely isolated TG neurons with diameters of 25–38 µm are classified into medium-sized groups [11,12]. Further, the medium-sized TG neurons are also subclassified into types: 2, 4, 8, 9, and 13 TG neurons. Among them, types 4, 8, 9, and 13 are classified as A neurons [11,12]. Therefore, most of the cells showing immunopositivity to Gnas and each receptor were medium-sized TG neurons that might be classified as A neurons; however, we could not distinguish the pure population of neurons from all the TG cells isolated morphologically. Our results also, showed that primary cultured TG neurons were immunopositive for NF-H, a neuron marker that is colocalized with the Gα_s_ subunit, indicating that at least medium-sized “A” neurons express Gα_s_ protein-coupled receptors. Although further studies are needed to clarify whether Gα_s_ protein-coupled receptors are expressed in specific neurons such as A neurons, our results indicated that β_2_, CGRP, A_2A_, D1, IP, and 5-HT_4_ receptors were expressed in the medium-sized NF-H-positive A neurons. We could not exclude the functional expression of these receptors on small-sized C neurons, however. The longest diameters of CALCRL-immunopositive cells ranged from 13.8 µm to 46.9 µm. The average of the longest diameter of CALCRL-immunopositive cells was 23.2 ± 8.7 µm. The average of the longest diameter was smaller than those of Gnas and each receptor-immunopositive cell. The small-sized acutely isolated TG neurons were subclassified into types 1–3 and 7 [11,12]. TG neurons of types 1, 2, and 7 represent C neuron properties [11]. Our results suggest that CGRP receptors may be expressed not only in A but also in C neurons. It has been reported that CGRP receptors are expressed in both Aδ and C neurons [13]. The reports are in line with our results from previous studies. Here, the classification of expression patterns of these receptors on small-, medium-, and large-sized TG neurons was of immediate interest.

In recent studies, it has been demonstrated that β_2_ receptors play a critical role in opioid tolerance, physical dependence, and opioid-induced hyperalgesia in rodent models [14,15]. Samoshkin et al. have reported that the opioid-induced Ca^2+^ response is dependent on β_2_ receptor activity in mouse TG neurons [16]. This suggests that β_2_ receptor activity is involved in opioid tolerance. This study revealed that TG neurons functionally expressed β_2_ receptors. These results imply that regulation of β_2_ receptor activity is involved in opioid treatment; however, further studies are needed.

The CGRP family of peptides has a widespread distribution of expression throughout the body, with particular abundance in the brain, gastrointestinal system, and various parts of the circulation [17]. The CGRP receptor is composed of CALCRL and receptor activity-modifying protein 1 (RAMP1) [18]. CGRP receptors are widely expressed everywhere in the brain, including in the TG and intracranial arteries [19]. CGRP-positive neurons are mostly small- to medium-sized neurons and also unmyelinated neurons, which are indicative of the cell bodies of sensory C fibers [20]. In the trigeminal nerve, CGRP is released from the nerve endings of C fibers and it is thought that the medium-sized Aδ fibers, in which the CGRP receptors are expressed, transmit pain signals to the central nervous system [21]. In accordance, our results showed that NF-H-positive TG cells are Gα_s_ protein-positive. In our previous study, we reported CGRP release from peptidergic C neurons of TG, but not NF-H-positive neurons, into the extracellular space following mechanical stimulation of the neurons [22].

ATP is a nociceptive mediator that is rapidly hydrolyzed to adenosine on the cell surface by ectonucleotidase on the cell membrane surface [23,24]. Adenosine is an endogenous nucleoside that ubiquitously resides throughout the body as an intermediary metabolite. Adenosine and the different types of adenosine receptors mediate a variety of physiological effects and participate in the maintenance of homeostasis in the nervous system [25,26]. Adenosine receptors are classified into four subtypes: A_1_, A_2A_, A_2B_, and A_3_ [27]. Different receptor subtypes are activated under different physiological or pathological conditions and adenosine concentrations. By controlling the neurotransmitter release and the action of neuromodulators, A_2A_ receptors can affect neuroinflammation, synaptic plasticity, and homeostasis in the nervous system [28]. In addition, it is reported that A_2A_ receptor activation is most likely to enhance pain responses in a pressure hyperalgesia model and the low-concentration formalin model [29].

In the mammalian brain, the major catecholamine neurotransmitter is dopamine [30]. Dopamine acts on dopamine receptors. The receptors are classified into two groups: D1-like (D1 and D5) and D2-like (D2, D3, and D4) receptors [31]. All of the dopamine receptors are GPCRs. The signaling of GPCR is mainly controlled by interactions with heterotrimeric GTP-binding proteins and activation of the proteins [31]. All dopamine receptors, except the D3 receptor, are expressed in the trigeminocervical complex [32]. Animal studies have suggested that the inhibitory effect of dopamine in animal models of persistent pain is mediated by D1 and D2 receptors [33]. Shamsizadeha et al. have reported that activation of D1-like receptors considerably reduces formalin-induced pain in rats and has an antinociceptive effect mainly on inflammatory pain in the CA1 region of the hippocampus, which participates in the process of pain sensation [34,35,36,37]. In the present study, TG neurons were found to be D1-like receptor agonist-sensitive and immunopositive for the D1 receptor, indicating that TG neurons functionally expressed D1 receptors. In addition, we observed the functional expression of A_2A_ receptors in TG neurons. Although A_2A_ and D1 receptors in TG neurons may also participate in chronic or acute pain processing, further studies are needed to completely understand the roles they play.

Arachidonic acid metabolites, such as prostaglandins (PGs), thromboxanes (TXs), hydroxy-eicosatetraenoic acids (HETEs), and leukotrienes (LTs), play unique and important roles in regulating various biological functions. Among them, prostanoids have been reported to participate in inflammation, and their application regenerated the predominant signs of inflammation, including augmented pain [38]. Davies et al. identified particular prostaglandins, principally prostaglandin E_2_ (PGE_2_) and prostacyclin (PGI_2_), as mediating vascular permeability and participating in edema and hyperemia caused by acute inflammation [39]. PGI_2_ also potentiates bradykinin-induced hyperalgesia [40,41]. In addition, inhibition of A kinase, which is activated by intracellular cAMP, suppresses hyperalgesia and increases intracellular cAMP levels caused by adenylyl cyclase activation, and PDE inhibition worsens hyperalgesia [42]. These results indicate that elevations in intracellular cAMP levels cause hyperalgesia through the activation of A kinase [4,42,43]. In this study, we showed that intracellular cAMP levels were increased by IP receptor activation in TG neurons. Whether activation of IP receptors in TG neurons enhances bradykinin-induced hyperalgesia via intracellular cAMP signaling is of immediate interest.

5-HT acts as an important neurotransmitter in the peripheral and central nervous systems. 5-HT receptors are classified into seven families and 13 subtypes [44]. All 5-HT receptors are GPCRs, except for the 5-HT_3_ receptors which are ligand-gated ion channels [45]. 5-HT promotes activation of tetrodotoxin-resistant Na^+^ channels via stimulating A kinase in dorsal root ganglion neurons, which might be capable of participating in the generation of hyperalgesia, while 5-HT is known to reduce glutamate release from primary-afferent central terminals in superficial dorsal horn neurons, resulting in the modulation of nociceptive transmission [46,47]. Thus, 5-HT and its receptor axis may be involved in sensory and pain processing pathways; however, to reveal this, further studies are needed.

## 5. Conclusions

We demonstrated that primary cultured TG neurons functionally expressed Gα_s_ protein-coupled β_2_, CGRP, A_2A_, D1, IP, and 5-HT_4_ receptors that regulate the intracellular cAMP signaling pathway. In addition, primary cultured TG neurons were immunopositive for NF-H, which is colocalized with the Gα_s_ subunit, indicating that medium-sized A neurons express Gα_s_ protein-coupled receptors. While our immediate interest lies in understanding how these Gα_s_ protein-coupled receptors and the subsequent intracellular cAMP signaling pathway modulate the nociceptive processing pathway, this comprehensive analysis of Gα_s_ protein-coupled receptor expression in TG neurons sheds light on potential mechanisms underlying nociceptive modulation in the orofacial region.

## Data Availability

All data is contained within the article.
